# Light-induced pyroelectric effect as an effective approach for ultrafast ultraviolet nanosensing

**DOI:** 10.1038/ncomms9401

**Published:** 2015-09-25

**Authors:** Zhaona Wang, Ruomeng Yu, Caofeng Pan, Zhaoling Li, Jin Yang, Fang Yi, Zhong Lin Wang

**Affiliations:** 1 School of Materials Science and Engineering, Georgia Institute of Technology, Atlanta, Georgia 30332-0245, USA; 2 Department of Physics, Applied Optics Beijing Area Major Laboratory, Beijing Normal University, Beijing 100875, China; 3 Beijing Institute of Nanoenergy and Nanosystems, Chinese Academy of Sciences, Beijing 100083, China

## Abstract

Zinc oxide is potentially a useful material for ultraviolet detectors; however, a relatively long response time hinders practical implementation. Here by designing and fabricating a self-powered ZnO/perovskite-heterostructured ultraviolet photodetector, the pyroelectric effect, induced in wurtzite ZnO nanowires on ultraviolet illumination, has been utilized as an effective approach for high-performance photon sensing. The response time is improved from 5.4 s to 53 μs at the rising edge, and 8.9 s to 63 μs at the falling edge, with an enhancement of five orders in magnitudes. The specific detectivity and the responsivity are both enhanced by 322%. This work provides a novel design to achieve ultrafast ultraviolet sensing at room temperature via light-self-induced pyroelectric effect. The newly designed ultrafast self-powered ultraviolet nanosensors may find promising applications in ultrafast optics, nonlinear optics, optothermal detections, computational memories and biocompatible optoelectronic probes.

## Abstract (web summary)

Zinc oxide is potentially a useful material for ultraviolet detectors, but a relatively long response time hinders practical implementation. Here, the authors demonstrate a zinc oxide nanowire/perovskite heterostructure detector with a five orders of magnitude improvement in both rising and falling edge time.

## Graphical abstract

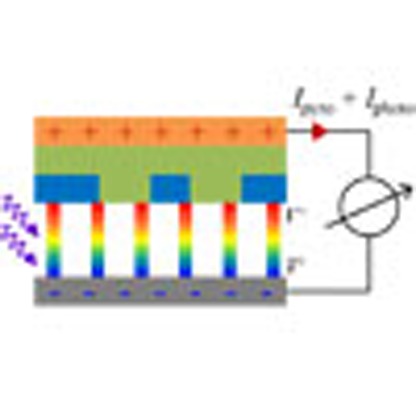

As one of the most widely used wurtzite semiconductor materials, ZnO has been extensively investigated as building blocks for nano/microdevices in various applications, including energy harvester^1^, optoelectronics^2^,^3^, field-effect transistors^4^ and logic computations^5^,^6^. Among these applications, room temperature ultraviolet detection is drawing tremendous attentions for defence technology, flame warning and chemical/biological analysis^7^,^8^ because of the wide bandgap (3.3 eV) and high exciton-binding energy (60 meV) of ZnO (refs 9, 10, 11, 12). Although numerous ZnO-based ultraviolet photodetectors (PDs) have been demonstrated with high photoresponsivity and/or specific detectivity, the relatively long response time and fluctuations in performance have always been an inevitable obstacle to improving the general performances of these PDs for practical applications. For example, the typical rising and falling times of previously reported ZnO-based ultraviolet PDs are ranging from a few seconds to few hours in most cases^13^,^14^,^15^,^16^,^17^. Therefore, it is necessary and essential to develop ultrafast response ultraviolet sensing devices based on ZnO micro/nanostructures.

Owing to the non-central symmetric crystal structures of wurtzite ZnO, pyroelectric potentials are induced by temperature changes across the material^18^,^19^,^20^. On light illuminations, a rapid rise in temperature is naturally induced within ZnO nanowires (NWs), leading to a distribution of pyroelectric potentials along the crystal with polarization pyrocharges presenting at both ends of the NW. A new approach is thus introduced to modify the charge–carrier transport process during the optoelectronic processes within ZnO-based devices by utilizing the pyroelectric polarizations.

In this work, we utilize this light-self-induced pyroelectric effect in ZnO to modulate the optoelectronic processes and thus enhance the performances of ultraviolet sensing. To maximize the enhancements of pyroelectric effect, low background temperature is maintained by minimizing the dark current (<0.1 nA) via designing and fabricating a self-powered PD that operates under zero bias^8^,^21^,^22^. Considering the simple synthesizing process and intrinsic optoelectronic properties, such as direct bandgap, large absorption coefficient and high carrier mobility^23^,^24^,^25^,^26^,^27^,^28^,^29^, CH_3_NH_3_PbI_3_ (MAPbI_3_) perovskite is applied to form a heterostructure with ZnO as self-powered PDs. The response time of the self-powered ZnO/perovskite-heterostructured (ZPH) PD is improved from 5.4 s to 53 μs in the rising time, and 8.9 s to 63 μs in the falling time by five orders in magnitudes. The specific detectivity D* and the responsivity *R* of the PD are both enhanced by 322%. The light-self-induced pyroelectric effect is attributed to these enhancements, and the working mechanism is carefully investigated. This work provides a novel design approach to achieve ultrafast response ultraviolet-sensing devices at room temperature enhanced by pyroelectric effect. The demonstrated self-powered PDs may become prospective candidates for ultrafast and energy-efficient ultraviolet sensing in different research and commercial fields.

## Results

### Design mechanism of self-powered ZPH PDs

**Figure 1:**
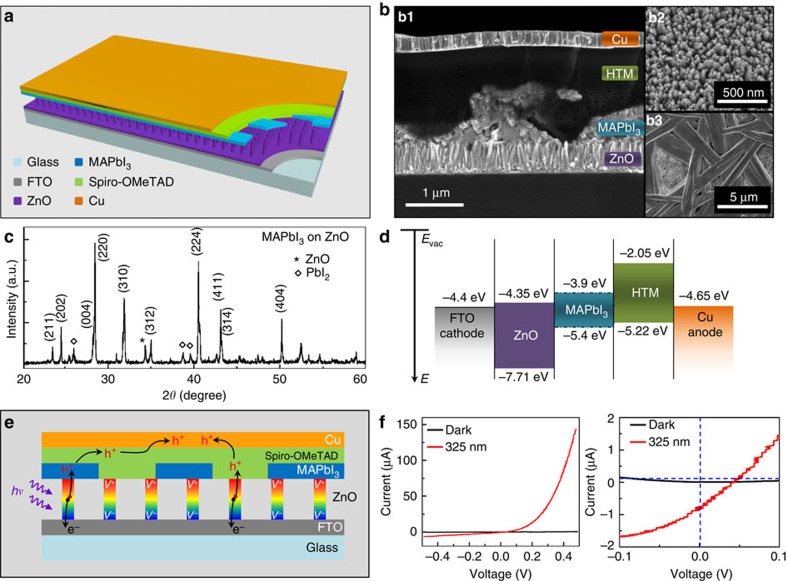
Structure, characterization and design mechanism of self-powered ZPH PDs. (**a**) Schematic demonstration of the structure of self-powered ZPH PDs. FTO acts as the transparent electrode. (**b**) Scanning electron microscopy images of self-powered ZPH PDs: (b1) side view of ZnO coated by perovskite, hole transport material (Spiro-OMeTAD) and Cu electrode in sequence (b2,b3) top view of ZnO NWs array (b2) before and (b3) after being spin-coated by perovskite. (**c**) XRD spectra of the perovskite (MAPbI_3_) on the ZnO NWs layer. (**d**) Energy band diagram of a self-powered ZPH PD. Energies are expressed in electron volts, using the electron energy in vacuum as a reference. The energy levels of the conduction band edges of ZnO, MAPbI_3_ and spiro-OMeTAD are at –4.35, –3.9 and –2.05 eV, respectively, and the valence band edge of the perovskite is at –5.4 eV. (**e**) Schematic illustration of the working mechanism of self-powered ZPH PDs. (**f**) *I–V* characteristics of the self-powered ZPH PDs under dark and 325-nm laser illumination with a power density of 3.7 mW cm^−2^.

The structure of self-powered ZPH PD is schematically illustrated in Fig. 1a. Side-view and top-view scanning electron microscopy images presented in Fig. 1b confirm the PD configuration, showing fluorine-doped tin oxide (FTO) glass uniformly and compactly covered by ZnO-NW array with diameters of 50–70 nm and lengths of 500 nm (top right panel of Fig. 1b, Supplementary Fig. 1a and Supplementary Note 1), and then partially covered by MAPbI_3_ perovskite with an average thickness of 500 nm (bottom right panel of Fig. 1b, Supplementary Fig. 1b and Supplementary Note 1), followed by a complete cover of hole transport material (HTM) spiro-OMeTAD with thickness of 500–1,000 nm (Supplementary Fig. 1c and Supplementary Note 1) and Cu top electrode with a thickness of 250 nm. The phase of MAPbI_3_ perovskite is verified by taking XRD spectra (Fig. 1c) of the perovskite film-covered ZnO-NW array^30^. Detailed fabrication processes and measurement set-up are found in the Methods.

Relevant energy levels of the materials used to fabricate the self-powered ZPH PDs are depicted in Fig. 1d. The valence band and conduction band of the MAPbI_3_ perovskite are −5.4 and −3.9 eV, versus vacuum, respectively^25^,^31^. On ultraviolet illumination, excitons are generated in the ZnO layer according to the absorption spectra of ZnO-NW array and ZnO/MAPbI_3_ perovskite film as shown in Supplementary Fig. 1d and in Supplementary Note 1. The energy band alignment of the device ensures electrons are injected from the photoexcited layer of the ZnO and collected by FTO electrode while the valence band holes reach the Cu electrode through the hole transport layer.

Ultraviolet illumination produces a fast temperature increase within pyroelectric ZnO NWs for the light absorption, due to the non-central symmetric crystal structures; pyroelectric potentials are induced across the NWs by polarizations, with positive potentials at the HTM/Cu electrode side and negative potentials at the FTO electrode side based on the *c* axis direction of ZnO-NW array, as shown in Fig. 1e. This light-self-induced pyroelectric potentials are aligning with the photovoltaic direction over the external circuit and thus enhance the transient short-circuit current and open-circuit voltage. More importantly, the pyroelectric potential presenting in ZnO NWs may speed up charge–carrier separation at the interface, preventing photoexcited electrons in ZnO from recombining with holes at the heterojunctions, further improving the performance of ultraviolet PD in response time. *I–V* characteristics of the self-powered ZPH PDs under 325-nm illumination (red curve in Fig. 1f) were measured and plotted in Fig. 1f in comparison with dark current (black curve in Fig. 1f), showing a good ultraviolet response of the device in output current, increasing from 1.5 μA (dark) to 143 μA (3.7 × 10^−3 ^W cm^−2^) when the HTM/Cu electrode was biased at 0.5 V. The self-powered operations are observed from the enlarged *I–V* curves (right panel of Fig. 1f) with a measurable (∼1 μA) photocurrent under ultraviolet illumination at zero bias. This can be attributed to the well-aligned band structure of the device (see Fig. 1d).

### General performances of the self-powered ZPH PDs

**Figure 2:**
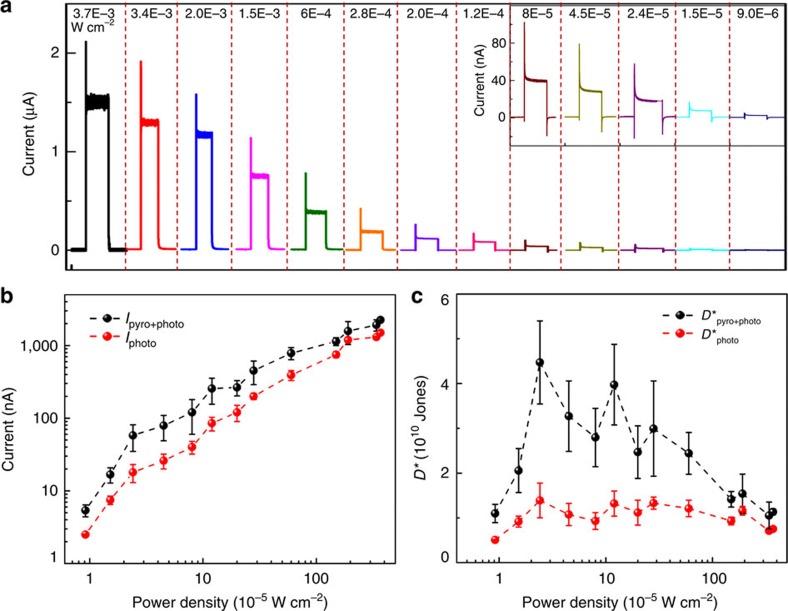
Pyroelectric effect enhanced performances of self-powered ZPH PDs. (**a**) *I–t* characteristics of self-powered ZPH PDs under 325-nm illuminations with different power densities from 3.7 × 10^−3^ to 9.0 × 10^−6 ^W cm^−2^, the inset is the enlarged *I–t* curves under the corresponding illumination conditions. (**b**) The short-circuit current response (**c**) and specific detectivity D* response of pyroelectric effect-combined photoexcitation process (black dots) and photoexcitation process (red dots), showing the enhancements by pyroelectric effect. Data reported in **b**,**c** were calculated from *I–t* curves acquired over 20 times within 30 days under different power densities.

The short-circuit current response of ZPH PDs to ultraviolet illumination was systematically investigated and summarized in Fig. 2a by varying the power densities from 3.7 × 10^−3^ to 9 × 10^−6 ^W cm^−2^. Under each power density, obvious two-stage short-circuit currents were derived. In the first stage, a sharp peak was induced by the combination of photovoltaic effect and pyroelectric effect on illumination which lead to an instantaneous temperature rise within ZnO. The corresponding short-circuit current is labelled as *I*_pyro+photo_; subscripts ‘pyro' and ‘photo' indicate the output current induced by pyroelectric effect and photovoltaic effect, respectively. In the second stage, the pyroelectric potentials disappeared as the temperature stayed constant by retaining the ultraviolet illumination, and the short-circuit current reached a steady plateau until shutting the laser beam, labelled as *I*_photo_. The corresponding currents of both stages were extracted and plotted in Fig. 2b as black and red dots, respectively, to demonstrate the pyroelectric enhancements on output signals of ZPH PDs. It is clear that the short-circuit currents *I*_pyro+photo_ and *I*_photo_ monotonically increase with the power density of illumination in both stages, with the current of the first stage larger than that of the second stage for all illumination conditions.

To better understand the enhancements by pyroelectric effect on the performances of ZPH PDs, specific detectivity D* is demonstrated in Fig. 2c for currents obtained from both stages, defined as D*=*R*/(2*e*·*I*_dark_/*S*)^0.5^ to describe the smallest detectable signal by considering the dark current *I*_dark_, which is in the order of 10^−11 ^A (Supplementary Fig. 3), as the major noise^32^. *S* is the effective area of the PDs, *R* is the corresponding photoresponsivity (Supplementary Fig. 2) calculated as 
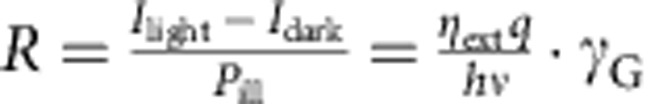
 (ref. 33), where *P*_ill_=*I*_ill_ × *S* is the illumination power on PDs; *I*_light_ represent the short-circuit current with illumination, respectively; *γ*_G_ is the internal gain; *η*_ext_ is the external quantum efficiency; *q* is electronic charge; *h* is Planck's constant; *v* is the frequency of the light; and *I*_ill_ is the excitation power density. Both D* and *R* derived from the first stage with a combination of pyroelectric effect and photovoltaic effect (labelled as D*_pyro+photo_) display larger values than those obtained from the second stage, where only photo-excitation process contributes (labelled as D*_photo_). The maximum value of D* is observed as 4.0 × 10^10^ and 1.3 × 10^10^ Jones for the first and second stages at the power density of 1.9 × 10^−5 ^W cm^−2^, respectively. Under the same condition, the maximum value of *R* is 26.7 and 8.3 mA W^−1^ for two stages (Supplementary Note 2). These results indicate 332% enhancements on both detectivity and photoresponsivity by pyroelectric effect.

The stability and repeatability of the self-powered ZPH PDs are studied and presented in Supplementary Figs 4 and 5 and in Supplementary Note 3. Short-circuit *I–t* curves of a typical device were measured under a power density of 3.7 × 10^−3 ^W cm^−2^ after keeping the device in air for 1 and 3 weeks (Supplementary Fig. 4), showing impressive stability of these PDs. Besides, the repeatability of the PD was also investigated by monitoring the *I–t* performances of four different devices fabricated through the same procedures as shown in Supplementary Fig. 5. Considering the self-powered operation mechanism of ZPH PDs, open-circuit voltage, similar to short-circuit current indicated above, can also be utilized to detect the illumination intensities as shown in Supplementary Fig. 6. The open-circuit *V–t* curves present a sharp peak followed by a stable plateau as well, with the open-circuit voltage in both stages increased monotonously with the power density (Supplementary Note 4).

### Working mechanism of the self-powered ZPH PDs

**Figure 3:**
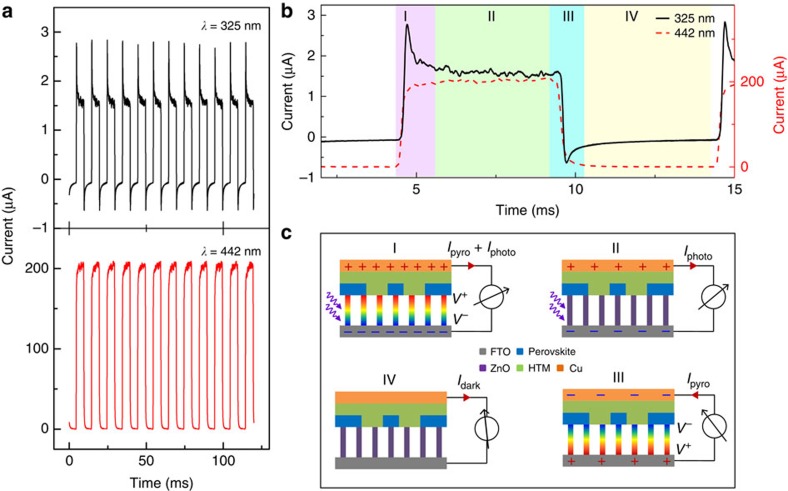
Working mechanism of self-powered ZPH PDs. (**a**) *I–t* characteristics of the self-powered ZPH PDs under 325 nm (top) and 442 nm (bottom) laser illuminations through an optical chopper with a time ratio of 1:1 at 100 Hz. (**b**) Enlarged plot of a single output period as shown in **a** for both 325- and 442-nm illuminations, divided into four stages, labelled as ‘I', ‘II', ‘III' and ‘IV'. (**c**) Schematic illustration of the working mechanism of pyroelectric effect-combined photoexcitation processes, corresponding to the four stages labelled in **b**.

Short-circuit *I–t* characteristics of the self-powered ZPH PDs were measured under 325 nm (top panel, Fig. 3a) and 442 nm (bottom panel, Fig. 3a) illuminations through an optical chopper with a time ratio of 1:1 at 100 Hz to investigate the physical mechanism of photon-sensing process combined with pyroelectric effect. On the basis of the absorbance spectra presented in Supplementary Fig. 1d, ZnO NWs are transparent to 442-nm illumination, and therefore the pyroelectric effect was only observed under 325 nm as clearly shown in Fig. 3a,b. The signal of 325-nm illumination displays a sharp rising edge, a stable plateau and a falling edge (black solid curve in Fig. 3b), while the signal of 442-nm illumination (red dash curve in Fig. 3b) is the same as those derived from traditional ZnO-based PDs reported previously^13^,^14^,^15^,^16^. Additional measurements were conducted to investigate the frequency dependence of self-powered ZPH PDs under both 325- and 442-nm illuminations. The corresponding *I–t* curves are presented in Supplementary Fig. 7, derived by varying the optical chopper frequency from 100 to 1,000 Hz. No obvious dependence on frequency was observed under 325-nm illumination, suggesting a potential ultrafast response of PDs enhanced by pyroelectric effect. As a comparison, the output signals were clearly affected by the frequency of light source under 442-nm illumination, indicating a slow response of these PDs without pyroelectric enhancements (Supplementary Note 5).

One cycle of the short-circuit *I–t* curve under 325 nm was divided into four stages, labelled as I, II, III and IV in Fig. 3b,c, to elaborate the physical mechanism of the pyroelectric effect-combined photon-sensing performances of self-powered ZPH PDs. In stage ‘I', the 325-nm ultraviolet illumination generates free carriers as photocurrent to transport through the external circuits from Cu to FTO electrode; meanwhile, an instantaneous temperature increase inside ZnO is produced on ultraviolet illumination, leading to a distribution of polarization pyroelectric charges across the NW with positive pyro-potential at the HTM/Cu electrode side, and negative pyro-potential at the FTO electrode side. The light-self-induced pyro-potential follows the photocurrent direction in external circuits and thus enhances the output signals with a sharp rising edge (I, Fig. 3b,c). In stage ‘II', the illumination is retained and the temperature stays at constant. Therefore, the pyroelectric potential disappears rapidly (in less than 1 ms) due to the leakage, and the output current reaches a stable plateau (II, Fig. 3b,c). In stage ‘III', the illumination is eliminated and the photocurrent thus disappears, while an instantaneous temperature decrease induced during this process results in a pyro-potential distribution in the opposite direction to that in ‘I', leading to current flows from FTO to Cu electrode as a falling peak with negative current magnitudes (III, Fig. 3b,c). In stage ‘IV', the temperature falls back to room temperature and stays steady; thus, the pyro-potential vanishes again because of leakage and the output returns to dark current (IV, Fig. 3b,c).

**Figure 4:**
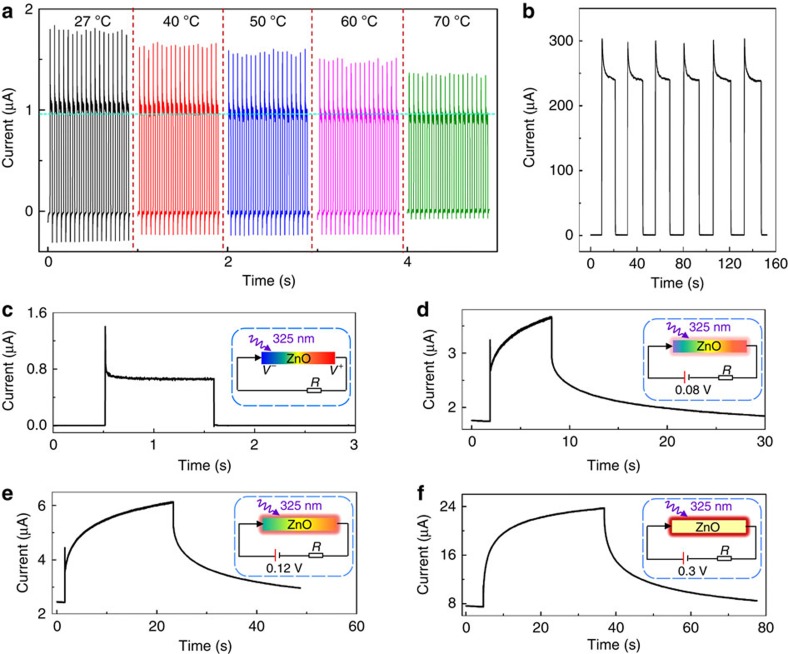
Pyroelectric effect enhancements on response time of self-powered ZPH PDs. (**a**) *I–t* response of the self-powered ZPH PDs to 325-nm illumination (power density of 1.8 mW cm^−2^) measured under different background environmental temperatures to confirm the pyroelectric effect. (**b**) *I–t* curves of the p-Si/ZnO PD biased at –2.0 V under 325-nm laser illumination with a power density of 2.6 × 10^−4 ^W cm^−2^. (**c**–**f**) *I–t* curves of the ZPH PDs under different bias voltages of (**c**) 0.0 V, (**d**) 0.08 V, (**e**) 0.12 V and (**f**) 0.3 V to calculate the improved response time by pyroelectric effect. The inset indicates the corresponding circuit diagram with current heating effect (red glow around ZnO) and pyroelectric effect of ZnO (colour code of ZnO).

The heating process of ultraviolet illumination was simulated by using finite element method based on the transient heat conduction equation as demonstrated in Supplementary Note 6. The temperature profiles, temperature variation rate d*T/*d*t* of the ZnO NW and the corresponding output currents induced by the pyroelectric effect were calculated and presented in Supplementary Fig. 8a,b. Furthermore, a nanosecond-pulsed laser with wavelength of 355 nm was applied to experimentally verify the pyroelectric response of ZPH PD as shown in Supplementary Fig. 8c,d. These theoretical simulations and experimental results provide convinced proofs to establish the direct correlation between observed optoelectronic performances and the ultraviolet illumination-induced pyroelectric effect in ZnO NWs (Supplementary Note 6). More control experiments were conducted to confirm the role played by the pyroelectric effect on the photon-sensing performances of the self-powered ZPH PDs. Figure 4a presents the short-circuit current response of the PDs under 325-nm illumination (power density of 3.7 × 10^−3 ^W cm^−2^) in different background environmental temperatures ranging from 27 to 70 °C. The sharp peak of the short-circuit currents related to pyroelectric effect *I*_pyro+photo_ decreased with increasing the environmental temperature, while the stable plateau of the current controlled by photo-excitation process *I*_photo_ remained unchanged. These results further verify the mechanism of light-self-induced pyroelectric effect in ZnO NWs. Moreover, by removing the perovskite layer, a new configuration of Si/ZnO PD was fabricated and tested as shown in Fig. 4b. Similar *I–t* characteristics (Fig. 4b) were obtained under 325-nm illumination, confirming the pyroelectric effect was induced within ZnO instead of other materials.

### Pyroelectric effect improved response time of ZPH PDs

Pyroelectric effect enhancements on response time of ZPH PDs were carefully studied by comparing the performances of devices operating under different bias voltages (0, 0.08, 0.12 and 0.3) V as shown in Fig. 4c–f. Higher applied bias voltage induced larger dark currents, which in turn increased the background temperatures of the device and thus reduced the pyro-potential in ZnO NWs as shown as the insets in Fig. 4c–f. *I–t* characteristic presented in Fig. 4c–f obviously indicates the gradual disappearance of pyroelectric effect-induced sharp peak as increasing the bias voltage. A double-exponential function^10^ fitting of the *I–t* curves was performed to calculate the response time of ZPH PDs under 0 and 0.3 V bias voltage as summarized in Supplementary Note 7 and Supplementary Fig. 9, with the corresponding fitting parameters shown as the insets. The rising time and the fall time are 53 μs (Supplementary Fig. 9b) and 63 μs (Supplementary Fig. 9c) for the self-powered ZPH PDs under zero bias, respectively. Considering the system response (∼28 μs) of the optical chopper switching the light signals, the actual response time of these PDs could be faster. Following the same fitting method, the rising time and fall time of these PDs operating under 0.3 V bias were calculated as 5.4 s (Supplementary Fig. 9d) and 8.9 s (Supplementary Fig. 9e), respectively. Therefore, the photon response time of ZPH PDs was reduced from several seconds to few microseconds (by 10^5^ time), by light-self-induced pyroelectric effect in ZnO NWs. Furthermore, these results also indicate essentiality to configure such PDs in a self-powered manner without applying any bias voltages to maximize the enhancements of pyroelectric effect.

## Discussion

These self-powered ZPH PDs possess novel, unique and complement features in comparison with conventional photosensing devices on the basis of top-down or bottom-up approaches. First, the long-overlooked pyroelectric effect induced by ultraviolet illuminations was employed to enhance the performances of photosensing. This is a fundamentally new mechanism that relies on the naturally existing temperature changes on ultraviolet illuminations. Compared with traditional methods^16^,^17^ to improve the response time and enhance the general performances of PDs, the pyro-phototronic approach demonstrated in this work is easier, faster and less costly. Moreover, this approach can be extended to other pyroelectric semiconductor-material combinations, including visible and/or infrared-sensitive materials, and thus broaden the spectra ranges for more practical applications. Second, self-powered PDs operated under zero bias were achieved by forming heterostructures between ZnO and perovskite. This configuration not only provides energy-efficient ultraviolet-sensing performances but also maintains low background temperatures by minimizing the dark current (<0.1 nA) to maximize the enhancements of pyroelectric effect. Third, the response time of these self-powered ZPH PDs was improved through pyroelectric effect from 5.4 s to 53 μs at the rising edge, and 8.9 s to 63 μs at the falling edge by five orders of magnitudes. The specific detectivity D* and the responsivity *R* of the PDs were both enhanced by 322%. The newly designed ultrafast ultraviolet PDs operating under zero bias may lead to further explorations in ultrafast optics, nonlinear optics, optothermal detections, computational memories and biocompatible optoelectronic probes.

## Methods

### Fabrication process of ZPH PDs

CH_3_NH_3_I was synthesized and purified following the previously reported method^34^. The synthesized CH_3_NH_3_I powder was mixed with PbI_2_ (Aldrich) at 1:1 mol ratio in N,N-Dimethylmethanamide (DMF) at 60 °C for 12 h, followed by filtering twice using 13-mm diameter and 0.45-mm pore polyvinylidene difluoride syringe filter (Whatman). The derived product was used as a coating solution for the formation of MAPbI_3_. Then, MAPbI_3_ perovskite DMF solution with 40 wt% was prepared for further usages. Meanwhile, the FTO glasses (Sigma-Aldrich, 13 Ω^−2^) were ultrasonically cleaned for 5 min in acetone, distilled water and ethanol, consecutively. Next, a layer of 50-nm ZnO seed layer was deposited on FTO glasses by radio frequency magnetron sputtering at room temperature (PVD75 system, Kurt. J. Lesker Company). The coated FTO glasses were then placed into the mixed nutrient solutions (0.02 M Zn(NO_3_)_2_ and 0.02 M HMTA) for ZnO NWs growth via a hydrothermal method in a mechanical convection oven (model Yamato DKN400, Santa Clara, CA, USA) at 85 °C for 30 min. To get separated ZnO NWs, 0–5 ml ammonium hydroxide (Sigma-Aldrich) was added per 100 ml mixing solution. After cooling down the whole system, the product was washed with ethanol and distilled water, collected and vacuum-dried at 100 °C for 1 h. A 40-wt% perovskite DMF solution was spin-coated on the dried ZnO array at 4,000 r.p.m., followed by spin-coating the spiro-OMeTAD as a hole transport thin layer with a spin coater (SCS 6800). Another thin layer of Cu (250 nm) was deposited as the bottom-electrode afterwards. Testing wires were connected to top and bottom electrodes by silver paste. Finally, a thin layer of kapton tape was employed to fix the testing wires and to improve its resistance to environmental contaminations and corrosions.

### Material characterizations

Detailed microscopic structures of ZnO NWs, perovskite, spiro-OMeTAD and copper film were characterized using scanning electron microscope (Hitachi SU8010), PANalytical X'Pert PRO diffractometer (Almelo, the Netherlands) with Cu KR radiation (*λ*=0.15418, nm) for X-ray diffraction patterns of the MAPbI_3_ perovskite.

### Optical and electrical measurements

Transmission spectra of ZnO and ZnO/perovskite were measured by a ultraviolet–visible spectrophotometer (JΛSCO V-630). *I–V* characteristics of the device were measured and recorded with a self-developed, computer-controlled measurement system with Stanford SRS Low-noise current preamplifier (SR570)/SRS low-noise voltage preamplifier (SR560) in conjunction with a GPIB controller (GPIB-USB-HS, NI 488.2). The optical input stimuli were provide by a He–Cd dual-colour laser (wavelength=325 and 442 nm, Model No. KI5751I-G, Kimmon Koha Co, Ltd). A continuously variable filter was used to control the light power density, which was measured with a thermopile powermeter (Newport 818 P-001-12). An objective was used to expand 325 and 442 nm laser to illuminate the whole device. The effective size of the device is 3 × 3 mm. A hot plate was used to control and change the background environmental temperatures.

## Additional information

**How to cite this article:** Wang, Z. *et al.* Light-induced pyroelectric effect as an effective approach for ultrafast ultraviolet nanosensing. *Nat. Commun.* 6:8401 doi: 10.1038/ncomms9401 (2015).

## Supplementary Material

Supplementary Information Supplementary Figures 1-9, Supplementary Notes 1-7 and Supplementary References
