# Supplementary material for: Light-induced pyroelectric effect as an effective approach for ultrafast ultraviolet nanosensing
Source: Nat Commun. 2015 Sep 25;6:8401. doi: 10.1038/ncomms9401 (PMC4598631; doi:10.1038/ncomms9401)
Supplement: Supplementary Information — Supplementary Figures 1-9, Supplementary Notes 1-7 and Supplementary References [file ncomms9401-s1.pdf]

Supplementary Figures

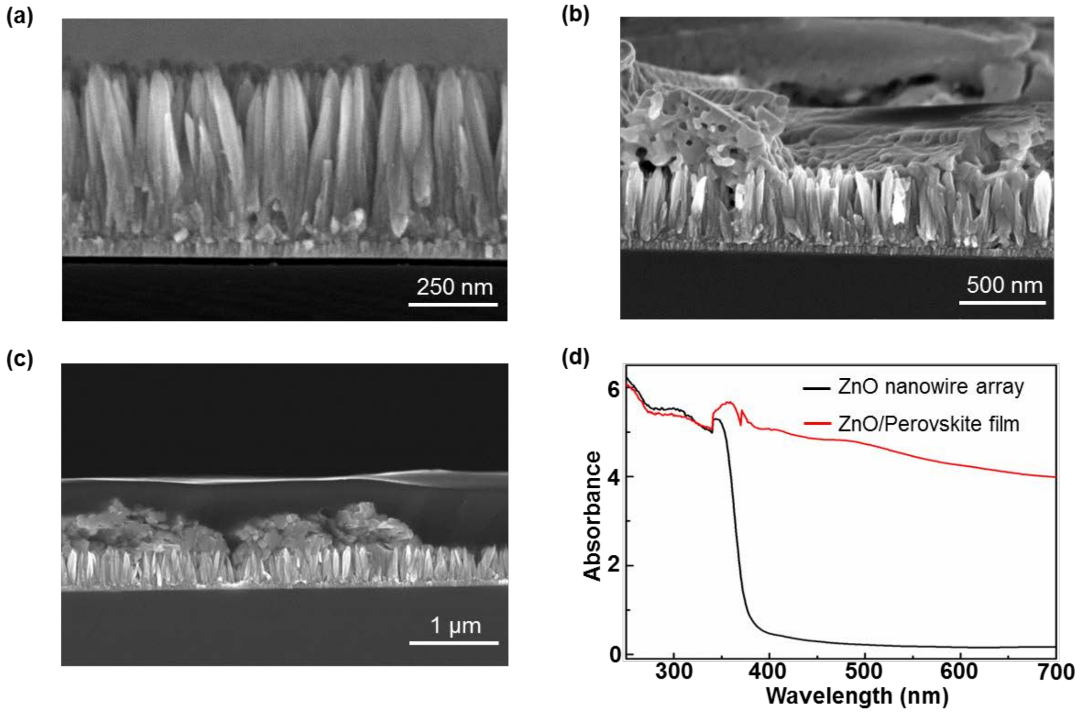

Supplementary Figure 1. Characterizations of self-powered ZnO/perovskite heterostructured photodetectors. (**a-c**) Scanning electron microscopy images of self-powered ZnO/perovskite heterostructured photodetectors: side view of ZnO nanowires array (a) before and (b) after coated by $MAPbI_3$ perovskite, (c) then coated by Spiro-OMeTAD layer. (**d**) Ultraviolet-visible absorption spectra of the ZnO nanowires array (in black) and the ZnO/perovskite film (in red).

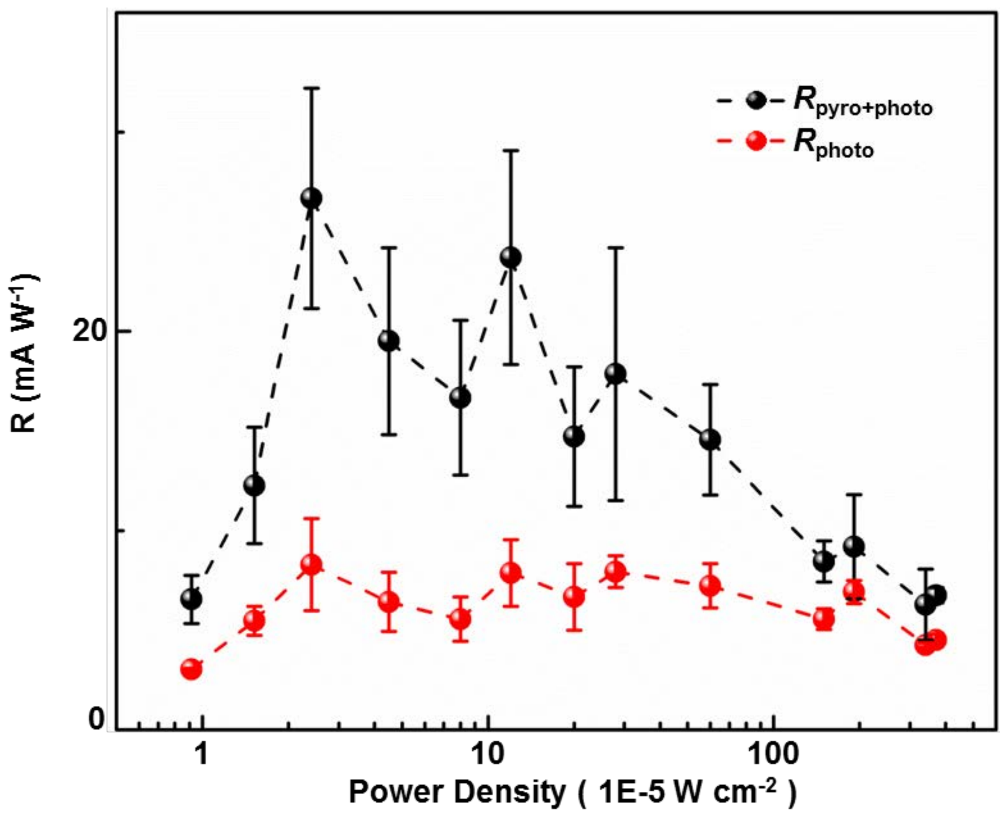

Supplementary Figure 2. Photoresponsivity of self-powered ZnO/perovskite heterostructured photodetectors. The photoresponsivity *R* response of pyroelectric effect combined photo-excitation process (black dots) and photo-excitation process (red dots), showing the enhancements by pyroelectric effect. Data reported in this figure were calculated from *I-t* curves acquired over 20 times within 30 days under different power densities.

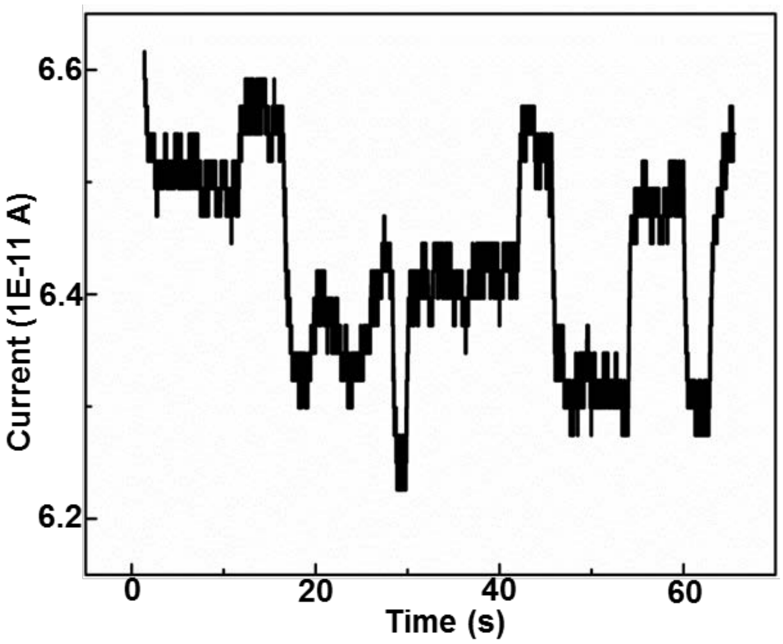

Supplementary Figure 3. Dark current of self-powered ZnO/perovskite heterostructured photodetectors.

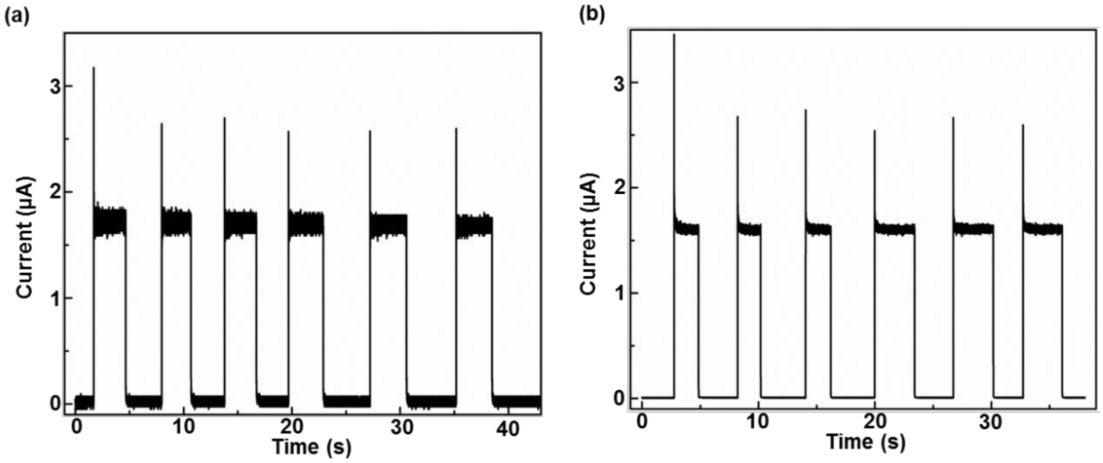

Supplementary Figure 4. Stability of the self-powered ZnO/perovskite heterostructured photodetectors. Short-circuit current *I-t* curves from the same device and under 325 nm illumination with the power density of 3.7×10^{-3} W cm^{-2} after the device was kept (a) one week (b) and three weeks in air.

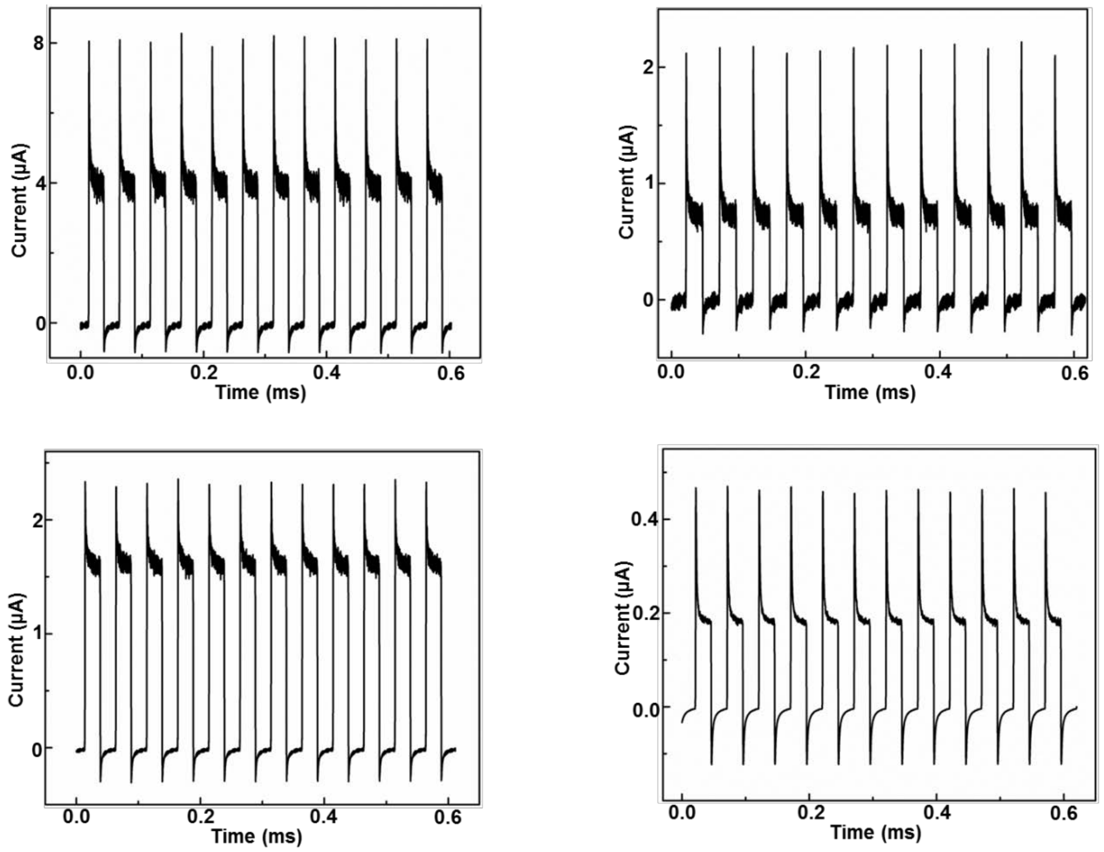

Supplementary Figure 5. Repeatability of self-powered ZnO/perovskite heterostructured photodetectors. Short-circuit current *I-t* curves from four different devices obtained through the same fabrication process and under 325 nm illuminations with the power density of 3.7×10^{-3} W cm^{-2}.

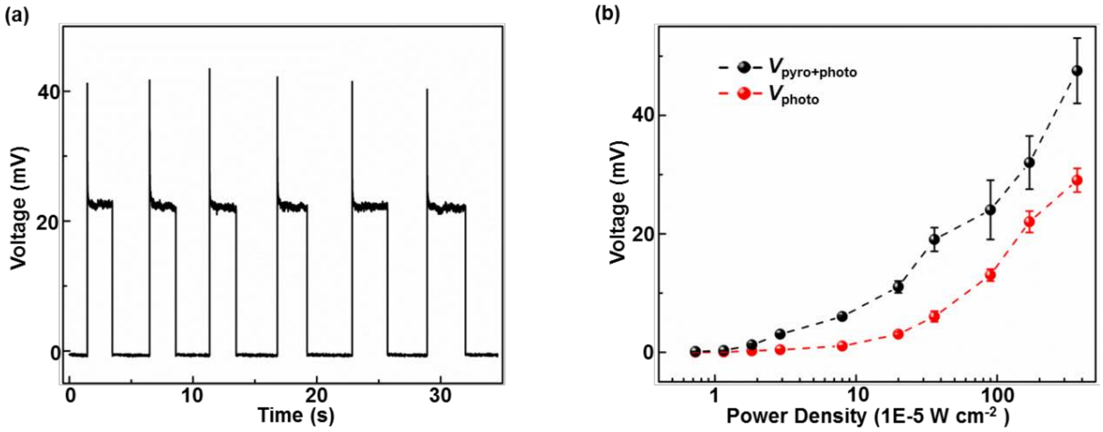

Supplementary Figure 6. Photoresponse of self-powered ZnO/perovskite heterostructured photodetectors in open-circuit voltage. (a) *V-t* curves of the

photodetectors under 325 nm laser illuminations with power density of 9.0×10^{-4} W cm^{-2}. (**b**) The open-circuit voltages response of pyroelectric effect combined photo-excitation process (black dots) and photo-excitation process (red dots), showing the enhancements by pyroelectric effect. Data reported in b were acquired over 10 times within 20 days under each power density.

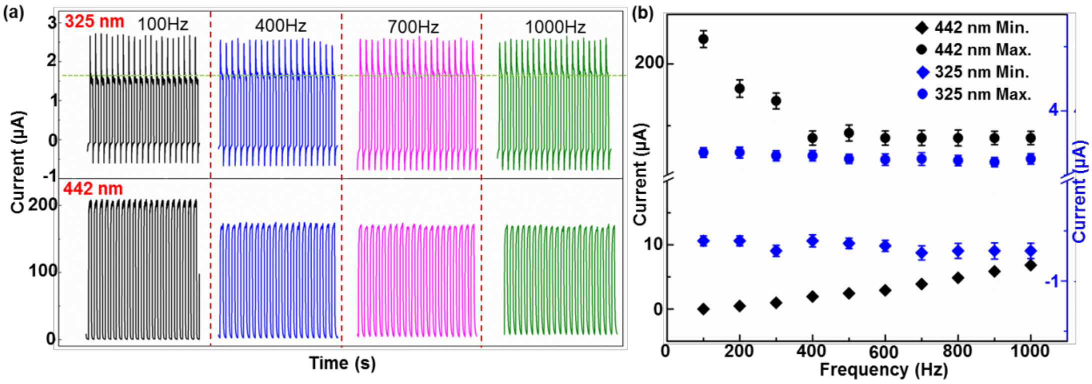

Supplementary Figure 7. Photoresponse of self-powered ZnO/perovskite heterostructured photodetectors under different frequencies. (**a**) *I–t* characteristics of the self-powered ZnO/perovskite heterostructured photodetectors under 325 nm (the top panel) and 442 nm (the bottom panel) laser illuminations at different frequencies (100-1000 Hz). (**b**) The maximum (in circle) and minimum (in diamond) values of short-circuit currents of the device illuminated by 325 nm laser (in blue) and 442 nm laser (in black) changes with the chopping frequency. Data reported in b were acquired over 10 times within 20 days under different frequencies.

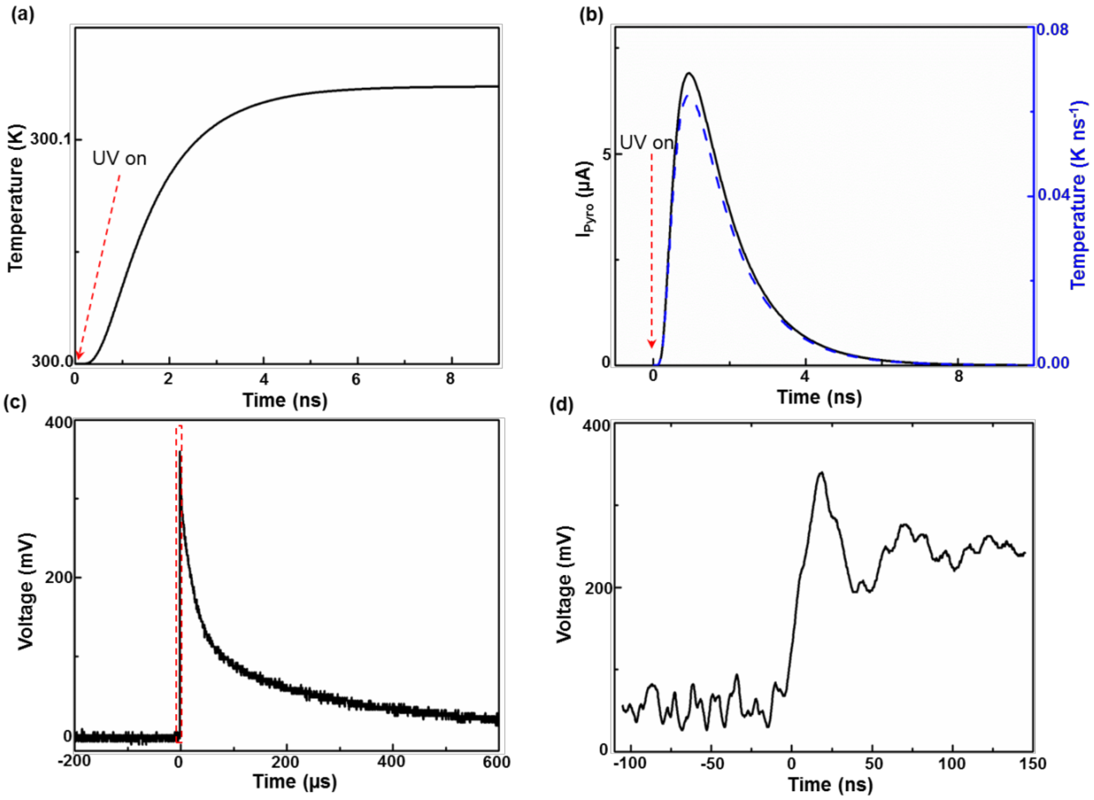

Supplementary Figure 8. **Pyroelectric effect of ZnO nanowires under ultraviolet illumination.** (**a**) Simulated temperature *vs*. time of ZnO nanowires at the heterojunction upon UV illumination. (**b**) The corresponding temperature variation rate (blue dash line) and the pyroelectrical effect-induced short circuit current (black solid line) of the device. (**c, d**)Time response of ZPH PD to the nano-second pulsed laser with wavelength of 355 nm. *V*-*t* curves in the timescale of (c) micro second and (d) nano second.

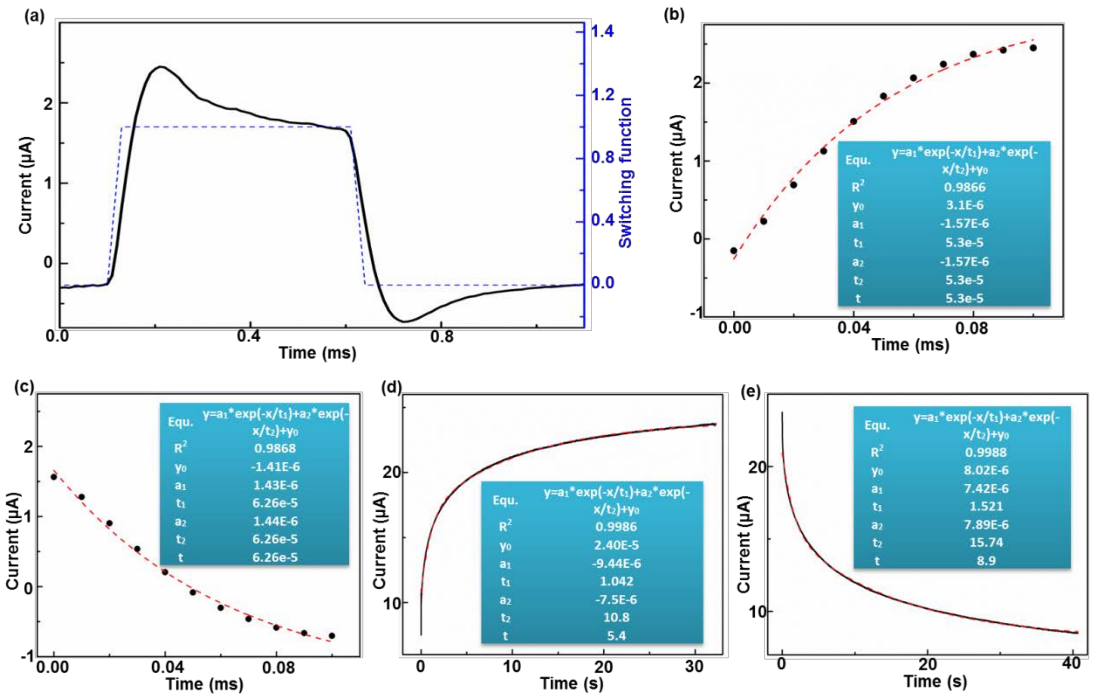

Supplementary Figure 9. Response time of self-powered ZnO/perovskite heterostructured photodetectors fitted by double-exponential functions. (a) *I*-*t* curves of the self-powered ZnO/perovskite heterostructured photodetectors at zero external bias (in solid line) and the switching function of the optical chopper (in dashed line) in the time scale of millisecond. (**b**) The rise and (**c**) fall time of PDs at zero bias; (**d**) the rise and (**e**) fall time of PDs at an external bias of 0.3 V.

Supplementary Notes

Supplementary Note 1 Characterization of self-powered ZnO/perovskite heterostructured photodetectors

Side-view scanning electron microscopy (SEM) images of the ZnO nanowire (NW) layer is demonstrated in Supplementary Figure 1a, showing the ZnO NWs vertically growing on the glass substrate and having a length of 500 nm. The aligned

configuration assures fast propagations of light through ZnO layer and thus a rapid response. Side-view SEM image of ZnO NWs/$MAPbI_3$ perovskite (Supplementary Figure 1b) shows that the ZnO NW array is partially covered by the perovskite layer. Supplementary Figure 1c shows the SEM image of ZnO/$MAPbI_3$/Spiro-OMeTAD, revealing the layer of Spiro-OMeTAD having a thickness of 500-1000 nm.

UV-visible measurements of both ZnO (black curve in Supplementary Figure 1d) and ZnO/$MAPbI_3$ heterojunction (red curve in Supplementary Figure 1d) synthesized on a glass substrate were conducted to indicate the spectrally-resolved response of our devices. The results show that the ZnO nanowires array and ZnO/$MAPbI_3$ heterojunction nanostructures can well respond to UV light (270 nm ~ 375 nm) and UV/visible light/infrared (270 nm ~ 700 nm), respectively. The ZnO/$MAPbI_3$ heterojunction used for UV-visible measurement are in the same structure and dimensions as we employed for self-powered ZnO/perovskite heterostructured photodetectors (ZPH PDs) fabrication.

Supplementary Note 2 Photoresponsivity of self-powered ZnO/perovskite heterostructured photodetectors

A critical parameter of photodetector, the photoresponsivity R is also calculated as shown in Supplementary Figure 2 for both stages of short-circuit photocurrents under 325 nm illuminations. The photoresponsivity R is defined as $R = \frac{I_{\text{light}} - I_{\text{dark}}}{P_{\text{ill}}} = \frac{\eta_{\text{ext}} q}{h\nu} \cdot \gamma_{\text{G}}$,[1] where $P_{\text{ill}} = I_{\text{ill}} \times S$ is the illumination power on PDs; I_{light} and I_{dark} represent short-circuit current with and without UV illumination,

respectively; γ_G is the internal gain; η_{ext} is the external quantum efficiency (EQE); q is electronic charge; h is Planck's constant; ν is the frequency of the light; I_{ill} is the excitation power density; S is the effective area of PDs. From Supplementary Figure 3, one can see that the dark current is in the order of 10^{-11} A, which is the major background noise attributed to the measurement system. Based on this value of dark current, photoresponse $R_{pyro+photo}$ and R_{photo} are calculated under 325 nm illuminationwith different power densities. A maximum value of 26.7 and 8.3 mA W^{-1} are derived for the photoresponse $R_{pyro+photo}$ (corresponding to transient current induced by pyroelectric and photo-excitation process) and R_{photo} (corresponding to stable current induced by the photo-excitation process) at the power density of 1.9×10^{-3} W cm^{-2}, respectively. It shows that the photonic responsivity is improved by 322% *via* the light-self-induced pyroelectric effect. The achieved R value is larger than that obtained from a Si/ZnO core-shell NW array PD (1.0×10^{-2} A W^{-1}, 480 nm, -1 V). [2]

Supplementary Note 3 Stability and repeatability of self-powered ZnO/perovskite heterostructured photodetectors

The stability of the self-powered ZPH PDs are shown in Supplementary Figure 4 by comparing the *I-t* curves from the same device and under the power density of 3.7×10^{-3} W cm^{-2} when the device was kept one week (Supplementary Figure 4a) and three weeks (Supplementary Figure 4b) in air. The photocurrents $I_{pyro+photo}$ and I_{photo} remained unchanged. It can be seen that the device demonstrate good stability.

Meanwhile, photon response characteristics of four different devices were also studied and shown as *I-t* curves in Supplementary Figure 5. The devices show similar *I-t* curves. The results show the stability and repeatability of our devices.

Supplementary Note 4 Open-circuit voltage response of self-powered ZnO/perovskite heterostructured photodetectors

The open-circuit *V-t* curves of the self-powered ZPH PDs is shown under 325 nm laser illuminations with the power density of 9.0×10^{-4} W cm^{-2} in Supplementary Figure 6. Similar curves to the short-circuit *I-t* curves in Figure 2a can be observed, as a sharp peak followed by a stable plateau when the laser was turned on or turned off, which can be used as a ultrafast write/read/erase pulses in the field of memory.[3] Defining the sharp peak as $V_{pyro+photo}$ and the stable plateau as V_{photo}, the open-circuit $V_{pyro+photo}$ and V_{photo} monotonously increase with the power density as shown in Supplementary Figure 6b, showing the optical intensity can also be determined by measuring the open circuit voltage.

Supplementary Note 5 Frequency-dependence of self-powered ZnO/perovskite heterostructured photodetectors

Additional measurements were conducted to investigate the frequency dependence of self-powered ZPH PDs under both 325 nm (upper panel, Supplementary Figure 7a) and 442 nm (lower panel, Supplementary Figure 7a) illuminations. The corresponding *I-t* curves are presented in Supplementary Figure 7a,

together with the *I-frequency* plots extracted from the *I-t* data as shown in Supplementary Figure 7b. The frequency of the optical chopper was varied from 100 to 1000 Hz during the measurement. Under 325 nm illuminations with pyroelectric effect enhancements, no obvious dependence on frequency was observed from the current response of the self-powered ZPH PDs, suggesting a potential ultra-fast response of PDs enhanced by pyroelectric effect. As a comparison, the output signals were clearly affected by the frequency of light source under 442 nm illuminations, indicating a slow response of these PDs without pyroelectric enhancements. There results confirm the enhancements on response time of self-powered ZPH PDs by light-self-induced pyroelectric effect.

Supplementary Note 6 Theoretical simulations and experimental measurements of the heating process upon UV illuminations

The heating process of UV illumination is simulated by using finite volume method based on the transient heat conduction equation. The temperature profiles and the corresponding temperature variation rate *dT/dt* of the ZnO nanowire at local ZnO/perovskite heterojunction interface are calculated and presented in Supplementary Figures 8a and 8b. Upon 355 nm UV illumination, the temperature of ZnO NWs increases exponentially and reaches the saturation point within a few nano seconds (Supplementary Figure 8a). The temperature variation rate *dT/dt* increases to the maximum in 2 ns and gradually decreases to zero as shown in Supplementary Figure 8b (blue dash line). Following the equation of pyroelectric current I =

p·A·(dT/dt), where *p* is the pyroelectric coefficient of ZnO nanowires as 1.2 nC cm^{-2}•K^{-1},[4] *A* is the effective area of the photodetector as 0.09 cm^2, the output currents induced by pyroelectric effect of the device are calculated and shown in Supplementary Figure 8b (black solid line). Similar to the trend of temperature variation rate *dT/dt*, the output currents also reach a maximum value in several nano seconds and then decrease to zero. These simulation results clearly indicate the presence of the pyroelectric effect in ZnO NWs upon illumination due to the naturally-existing heating effect, and the corresponding ultra-fast response to UV light of the devices. Furthermore, a nano second pulse laser with wavelength of 355 nm (Continuum model PowerLite Precision 8000) is applied to experimentally verify the pyroelectric response of ZPH PD as shown in Supplementary Figures 8c and 8d. The response time at rising edge is about 16 ns, while the recovery time is relatively long and characterized by the electric time-constant τ_E of the whole system.[5] These experimental data further confirm the theoretical simulation results and the essential role played by the pyroelectric effect of ZnO NWs through the optoelectronic processes for ultra-fast UV sensing.

Supplementary Note 7 Response time of self-powered ZnO/perovskite heterostructured photodetectors

I-t curves of the self-powered ZPH PDs without any external bias and the switching function of the chopper are presented in Supplementary Figure 9a in the timescale of millisecond. The fast response can be obtained. Here, to clearly show the

changes of response time at rise/fall edges with external bias, the best fit of data obtained by a double-exponential function[6, 7] is presented in Supplementary Figures 9b-e, with the corresponding fitting parameters shown in the insets.

Based on the fitting functions and parameters presented in the inset of Supplementary Figures 9b (rise) and c (fall), a weight-averaged rise time[6] of 53 μs and fall time of 63 μs are derived for self-powered ZPH PDs at zero bias. When applying an external bias of 0.3 V (in Figure 4f), a long rise time of 5.4 s and fall time of 8.9 s are obtained by applying the same fitting functions as presented in Supplementary Figures 9d (rise) and e (fall). These results indicate pyroelectric effect improves the response time of self-powered ZPH PDs. The light-self-induced pyroelectric potential significantly improves/reduces the response time at rise/fall edges.

Supplementary References

1. Konstantatos G, Sargent EH. Nanostructured Materials for Photon Detection. *Nat Nanotechnol* **5**, 391-400 (2010).

2. Sun K, Jing Y, Park N, Li C, Bando Y, Wang DL. Solution Synthesis of Large-Scale, High-Sensitivity ZnO/Si Hierarchical Nanoheterostructure Photodetectors. *J Am Chem Soc* **132**, 15465-15467 (2010).

3. Wu WZ, Wang ZL. Piezotronic Nanowire-Based Resistive Switches As Programmable Electromechanical Memories. *Nano Lett* **11**, 2779-2785 (2011).

4. Yang Y*, et al.* Pyroelectric Nanogenerators for Harvesting Thermoelectric Energy. *Nano Letters* **12**, 2833-2838 (2012).

5. Odon A. Processing of signal of pyroelectric sensor in laser energy meter. *Measurement*

Science Review **1**, 215-218 (2001).

6. Soci C*, et al.* ZnO nanowire UV photodetectors with high internal gain. *Nano Lett* **7**, 1003-1009 (2007).

7. Ni PN, Shan CX, Wang SP, Liu XY, Shen DZ. Self-Powered Spectrum-Selective Photodetectors Fabricated from n-ZnO/p-NiO Core-Shell Nanowire Arrays. *J Mater Chem C* **1**, 4445-4449 (2013).
